# Microtubule structure by cryo-EM: snapshots of dynamic instability

**DOI:** 10.1042/EBC20180031

**Published:** 2018-10-12

**Authors:** Szymon W. Manka, Carolyn A. Moores

**Affiliations:** Institute of Structural and Molecular Biology, Department of Biological Sciences, Birkbeck, University of London, London, U.K.

**Keywords:** cytoskeleton, cryo-electron microscopy, cryo-electron tomography, microtubule, MAPs, molecular motors

## Abstract

The development of cryo-electron microscopy (cryo-EM) allowed microtubules to be captured in their solution-like state, enabling decades of insight into their dynamic mechanisms and interactions with binding partners. Cryo-EM micrographs provide 2D visualization of microtubules, and these 2D images can also be used to reconstruct the 3D structure of the polymer and any associated binding partners. In this way, the binding sites for numerous components of the microtubule cytoskeleton—including motor domains from many kinesin motors, and the microtubule-binding domains of dynein motors and an expanding collection of microtubule associated proteins—have been determined. The effects of various microtubule-binding drugs have also been studied. High-resolution cryo-EM structures have also been used to probe the molecular basis of microtubule dynamic instability, driven by the GTPase activity of β-tubulin. These studies have shown the conformational changes in lattice-confined tubulin dimers in response to steps in the tubulin GTPase cycle, most notably lattice compaction at the longitudinal inter-dimer interface. Although work is ongoing to define a complete structural model of dynamic instability, attention has focused on the role of gradual destabilization of lateral contacts between tubulin protofilaments, particularly at the microtubule seam. Furthermore, lower resolution cryo-electron tomography 3D structures are shedding light on the heterogeneity of microtubule ends and how their 3D organization contributes to dynamic instability. The snapshots of these polymers captured using cryo-EM will continue to provide critical insights into their dynamics, interactions with cellular components, and the way microtubules contribute to cellular functions in diverse physiological contexts.

## Introduction

Since microtubules (MTs) were first observed in cells [1,2] and tubulin was first purified and proposed to be the building block of MT [3], electron microscopy (EM) has been a crucial technique for investigating MT molecular mechanism and functional context in cells and tissues. Early EM work helped define the organization of individual tubulin αβ-heterodimers, aligned head-to-tail within protofilaments (PFs) and linked via lateral connections between PFs to form the cylindrical, polar MT tube. Initial progress on MT ultrastructure–and the diversity of other oligomers and polymers that tubulin can form—was made using heavy metal staining [4–6]. However, the advent of cryo-electron microscopy (cryo-EM) [7] meant that MTs could be captured in their solution-like state [8], paving the way for decades of insight into their dynamic mechanisms and interactions with binding partners including molecular motors.

**Figure 1:**
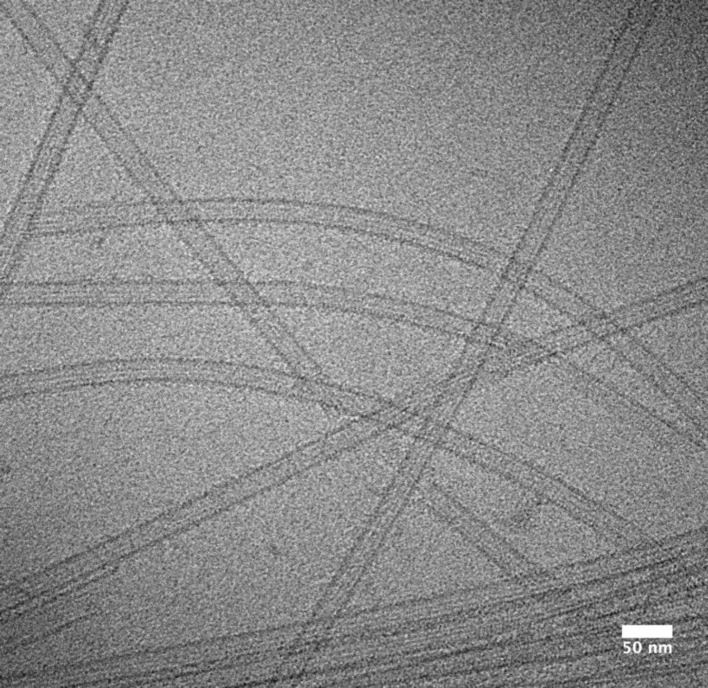
Unstained frozen-hydrated MTs stabilized with DCX and Taxol Electron cryo-micrograph was obtained with a 120 kV microscope (Tecnai T12, FEI). Straight and curved MTs with a uniform diameter (13-PF architecture) are darker than ice (vitrified buffer). Some polymers are bundled, flattened or otherwise disrupted. In intact MTs, PFs are visible by glancing along their length and their number can be deduced from the striation pattern (so called moiré pattern) [8]. MT edges appear darker due to the presence of several PFs in the projection.

Early cryo-EM micrographs provided compelling 2D visualizations of these beautiful polymers (Figure 1). The ultra-fast freezing (vitrification) in cryo-EM sample preparation not only revealed the appearance of the MT lattice, but also enabled the conformational variability of dynamic MT ends to be captured [9]. MT ends are the major sites of dynamic transitions—a behavior termed dynamic instability [10]—and interpretation of tubulin conformations visualized in these regions still challenges the field (see below).

MTs polymerized *in vitro* from mammalian brain-purified tubulin (still the most practical source of tubulin) are built from a range of PF numbers, typically from 11 to 16. This contrasts with the predominance of 13-PF MTs seen in most eukaryotic cells [4,11]. Although different polymerization conditions can alter this PF distribution [12], how this is determined and its significance is still not understood. Nevertheless, the diversity of polymer architecture has helped define the rules by which the lattice is built [13], highlighting the presence of a lattice discontinuity/discontinuities—known as seams—in which the otherwise helical arrangement of tubulin subunits within the MT lattice is interrupted.

As well as allowing direct visualization of MTs, the 2D images collected in cryo-EM experiments can be used to computationally reconstruct the 3D structure of the polymer. An important early landmark was determination of the 3D structure of the tubulin dimer itself, not from cryo-EM images of MTs in the first instance but from 2D crystals of zinc-induced sheets of anti-parallel PFs [14] (see below). This work defined the PF structure and the positions of the exchangeable (E-site) and non-exchangeable (N-site) GTP/GDP-binding sites within the tubulin dimer, subsequently allowing the PF configuration within MTs to be defined [15].

**Figure 2:**
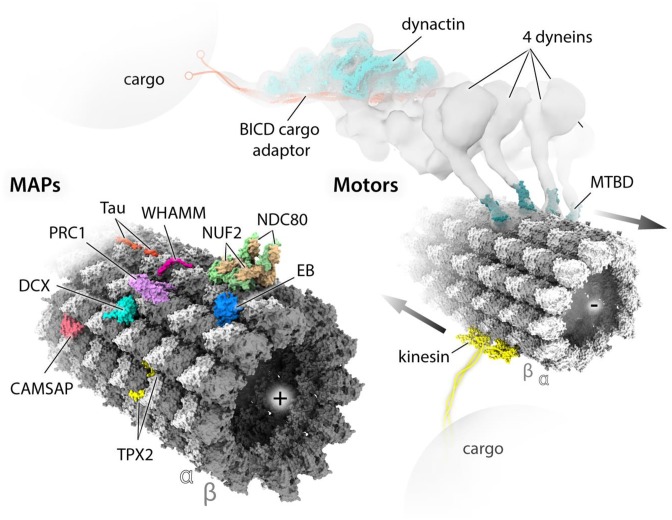
MAPs and motor proteins studied by cryo-EM Several structures of MTBDs or MT-binding polypeptide regions of MAPs and motor proteins have been determined in association with MT lattice by single-particle cryo-EM performed on decorated MTs. Atomic models of these structures are placed at relevant sites on the surface of an atomic model of MT lattice, all shown in solvent-excluded surfaces (SES) representation generated with ChimeraX [71]. The MT model was obtained by fitting a 6-dimer atomic model (PDB: 6EVZ) into near-atomic resolution cryo-EM map (EMDB: EMD-3964); the polarity of each MT is indicated by (+) and (−) signs. MAP models include: tau (PDB: 6CVN), WHAMM (PDB: 5X1G), NDC80/NUF2 (PDB: 3IZ0), EB (PDB: 3JAK), PRC1 (PDB: 5KMG), DCX (PDB: 4ATU), CAMSAP (PDB: 5M5C) and TPX2 (PDB: 6BJC). Motor models include two copies of an example typical (+) end directed kinesin motor domain (PDB: 5ND7) and four copies of the (−) end directed dynein MTBD (PDB: 3J1T) reconstructed together with MTs. The cluster of four dyneins assembled on dynactin and the MT was identified by cryo-ET [66], with lower resolution than the single-particle studies, which is indicated with isosurface representation of the experimental density (EMDB: EMD-7001). Structures of all dynein domains have been determined at high-resolution by both X-ray and cryo-EM studies not involving MT templates/tracks, hence not fitted into the density, except the dynactin/BICD part of the complex indicated for clarity. Kinesin motor domains are joined (dimerized) by the cartoon of a kinesin neck region and coiled-coil stalk region extending to cargo-binding elements. Similarly, BICD cargo adaptor is extended with a cartoon of cargo-binding elements.

The combination of cryo-EM imaging and 3D reconstruction has allowed binding site determination for numerous components of the MT cytoskeleton, including motor domains from many members of the kinesin superfamily of molecular motors, and the MT-binding domains (MTBDs) of dynein motors and a host of MT-associated proteins (MAPs) (Figure 2). In these experiments, the cylindrical geometry of a MT is exploited to determine the structure of a MTBD in its MT-bound conformation. Multiple copies of the MTBD of interest attach regularly along the lattice according to their particular binding site—e.g. kinesin motor domains bind every tubulin dimer, centered over the intra-dimer interface. The many views of the MT-bound MTBD present in each MT image are computationally averaged to reveal its 3D shape. In turn, this binding serves as a regular marker of the underlying α- and β-tubulin subunits within the lattice, thereby also revealing the position of the seam in the pseudo-helical MT architecture [16,17]. Recently, the availability of direct electron detectors has caused a resolution revolution in cryo-EM [18] and enabled the calculation of near-atomic reconstructions of a diversity of MT-binding protein complexes [17,19–24]. In tandem with software development [25], these data now enable the relatively subtle differences between α- and β-tubulin to be distinguished computationally, enabling near-atomic reconstructions of naked (undecorated) MTs [26], and of MTs decorated with unstructured or flexible MAPs, interacting with MT lattice via relatively short oligopeptides [22,24,27] (Figure 2).

These technical developments have also enabled visualization of the subtle structural transitions within the MT lattice driven by the tubulin GTPase activity and which drive dynamic instability [17,19,23,26]. These structural findings have provided vital insights into the molecular basis of dynamic instability, which is crucial for many MT functions. This includes the highly dynamic MT-based spindle machinery that drives cell division and which is targeted by widely used cancer chemotherapeutics. Here, we concentrate on *in vitro* studies of MTs using cryo-EM with a focus on molecular fundamentals of MT dynamics, and MAPs and drugs, whose activities modulate structural and functional characteristics of MTs.

## MAPs and motors on MT tracks

MAPs are essential in mediating the physical and biochemical properties of MTs with a wide range of cellular functions. Some of them modulate these properties *in vitro* and *in vivo*, for example, by spatially and temporarily regulating MT nucleation, dynamics, stability or post-translational modification patterns (‘tubulin code’) [28] at various stages of the cell cycle. Motor proteins, on the other hand, are required for various mechanical tasks, such as intra-cellular transport or flagellar beating, using energy from ATP binding and hydrolysis. Different MAP and motor MTBDs characterized by cryo-EM through co-polymerization or decoration of MTs are presented in Figure 2 and will be briefly discussed.

### MAPs with linear MT-binding motifs

Tau and its isoforms [29] were the first identified MAPs and, together with related MAPs such as MAP2, are largely unstructured. This has presented a long-running challenge to structural biologists to identify its MT-binding site. Lower resolution (∼30 Å) reconstructions yielded conflicting conclusions that tau/MAP2 bound on either the outer PF ridge [30] or in the MT lumen [31]. However, with direct detector data and more modern reconstruction algorithms, tau’s binding mode on the outer surface of MTs has been precisely confirmed at high resolution [22]. Conserved oligopeptide repeats of tau stabilize MTs by binding in tandem across tubulin dimers on the PF ridge (Figure 2). Another critically important interaction of tau with MTs involves the negatively charged C-terminal tails of tubulin (also called E-hooks) [32], but because they are disordered, they remain obscure to structural analyses by cryo-EM. Hyperphosphorylation of tau favors its dissociation from MTs and aggregation into amyloid fibers found in Alzheimer’s disease, the structures of which were also recently solved by cryo-EM [33].

TPX2 and WHAMM are examples of modular, multidomain and multifunctional MAPs. TPX2 is important for chromatin-mediated MT nucleation during the onset of mitosis [34]. It facilitates MT nucleation by binding the MT wall via two elements: the ‘ridge’ and ‘wedge’, which are flexibly linked in the middle region of the protein to simultaneously span longitudinal and lateral lattice contacts (Figure 2). Only the ‘ridge’ and ‘wedge’ are visible in the MT reconstruction due to flexibility of the rest of the TPX2 molecule. WHAMM is a WASP homolog associated with actin, membranes and MTs. It nucleates actin filaments and links them with MTs and membrane vesicles to remodel them into tubular structures upon transport from the endoplasmic reticulum to the Golgi apparatus [35,36]. Cryo-EM reconstructions revealed binding of an oligopeptide in the central coiled-coil region across α- and β-tubulin bridging two dimers in the MT lattice [27,37] (Figure 2), explaining how WHAMM’s activities are structurally coordinated.

### MAPs with globular MTBDs

Other MAPs bind to MTs via globular MTBDs. Intriguingly the calponin homology (CH) domain is shared among several different MAPs that bind MTs in quite different ways. End-binding proteins (EBs) autonomously and dynamically bind a region immediately behind growing MT (+) and (−) ends via their CH domain [38,39], an activity that causes the classic comet-like behavior of fluorescently labeled EBs in cells. EB CH domains bind preferentially to MTs grown in the presence of the GTP analogs GTPγS and GDP.BeF [40,41], suggesting that EBs track growing MT ends by recognizing a GDP.Pi-like structural state of tubulin within the MT lattice. The first pseudoatomic model of a CH domain bound to MTs was that of the fission yeast EB Mal3 determined at sub-nanometer resolution [40]. This structure revealed that the CH domain of Mal3 bridges PFs at the corner of four tubulin dimers, adjacent to E-sites of two β-tubulins, thereby suggesting a direct route for sensing the nucleotide state of MT lattice. More recently, a near atomic resolution reconstruction of the CH domain from the mammalian EB3 protein decorating GTPγS-MTs allowed atomic model refinement directly in the EM density, characterizing EB–MT interactions in more detail and shedding more light on GTPase-dependent conformational changes in tubulin (discussed more below) and the sensitivity of EBs to them [19].

Both NDC80 and NUF2—the MT-binding components of the NDC80 hetero-tetrameric kinetochore complex— contain a CH domain that can recognize both intra- and inter-dimer interfaces. Species specific variations have been reported [42], but the human NDC80 complex appears to bind MTs uniformly with a tubulin monomer repeat (Figure 2). The disordered cationic N-terminal tail of NDC80 is critical for high-affinity MT binding [43] and is the target of phospho-regulation by Aurora B kinase [44]. The sub-nanometer cryo-EM reconstructions of NDC80-decorated MTs revealed that this region mediates self-association of NDC80 complexes along PFs, shedding light on how they stay attached to spindle MTs while these MTs depolymerize, driving segregation of chromosomes during cell divisions [45–47].

The calmodulin-regulated spectrin-associated protein (CAMSAP)/Patronin family of proteins participates in organizing non-centrosomal MT networks important for cell division, polarization and differentiation by regulating MT (−) end dynamics [48,49]. The globular C-terminal CKK domain of CAMSAPs autonomously recognizes MT (−) ends and also binds to the MT lattice between two tubulin dimers at the inter-PF interface [50] (Figure 2), a binding site that lies 4 nm away from that of the EBs. Cryo-ET experiments provided insight into the 3D structure of the transition zone between the regular lattice and the more curved, sheet-like PFs at MT ends. The CKK MT (−) end preference was proposed to arise from CKK recognition of the intrinsic polarity by which tubulin curvature develops at MT ends [50].

The neuronal MT-nucleating and stabilizing protein doublecortin (DCX) contains two ubiquitin-like MTBDs separated by a 42-residue unstructured linker. It was shown to strongly promote 13-PF lattice architecture *in vitro* [51,52]. Via a single DC domain, DCX binds in the vertex of four tubulin dimers in the lattice [53] (Figure 2), the same binding site as EBs [40]. DCX stabilizes both longitudinal and lateral lattice contacts and is sensitive to the nucleotide state of the lattice [23]. The length of the linker between the DC domains would theoretically permit simultaneous binding of both N- and C-DC domains to the MT lattice, but due to their highly similar fold, current sub-nanometer resolution reconstructions have not yet clarified the extent of their individual involvement in this interaction.

The MTBD in PRC1 (protein regulator of cytokinesis 1) is elongated and projects from the MT wall [54]. It cross-links MTs by dimerizing via its N-terminal domains and binding to MTs via the C-terminal spectrin domains. This facilitates generation of anti-parallel MT arrays during mitosis. The spectrin domains bind on the crest of PFs like kinesins, each to a single tubulin dimer (Figure 2), determining the geometry of the resulting PRC1-MT arrays [21,55].

### Kinesins and dyneins

Kinesins and dyneins are complex, multidomain and multimeric motors that navigate around the MT cytoskeleton. Globular ∼40 kD kinesin motor domains bind MTs and undergo ATPase-linked conformational changes that drive motor movement (Figure 2). Cryo-EM studies of motor domains from a wide range of kinesins have yielded key insights into their mechanochemistry [56–61]. The dynein motor (ATPase) domain is completely unrelated to kinesin and is a 6 AAA (+) (‘ATPases associated with diverse cellular activities’) ring separated from the MT track by a long coiled-coil stalk ending with its relatively small (∼10 kD) MTBD (Figure 2). Cryo-EM structures of MT-bound dynein MTBD [62,63] have shown that its MT-binding site is very similar to that of kinesins and is centered over the intra-dimer interface of the tubulin dimer. Larger complexes of cytoplasmic or ciliary dyneins bound to MTs have been studied by tomography (cryo-ET) [64–67]. An emerging theme in the way that MT-based motor transport is regulated is the response of kinesins and dyneins to MAPs present on the motors’ MT tracks [68–70]. The reconstitution and structure determination of multicomponent motor, MAP-MTs, is an exciting future direction that offers structural insight into the cross-talk between various components of the MT cytoskeleton. Thus, we will gradually become equipped with more and more sophisticated tools to literally look at MT biology and its associated pathologies.

## Tubulin conformational landscape and structural basis of dynamic instability

Over three decades since the discovery of MT dynamic instability [10], a full understanding of its mechanism is still lacking. We know that it is driven by GTP hydrolysis in the β-tubulin subunit within the MT lattice, but it is unclear why the GTP lattice is stable and forms a cap that supports MT growth, while the GDP lattice is unstable, resulting in MT catastrophe when the cap is lost (i.e. when hydrolysis outpaces growth). Two sets of structural observations are likely important aspects of this behavior: (1) X-ray and cryo-EM studies have shown that tubulin adopts a bent conformation outside the context of the MT lattice [9,72,73], suggesting that the lattice-induced straight conformation generates mechanical strain balanced by contacts formed within the lattice [9,74–77] and (2) the MT lattice compacts longitudinally by ∼2 Å per dimer following GTP hydrolysis. This was first shown in mid 1990s [78,79], and recently visualized in detail by high-resolution cryo-EM: MTs bound by the slowly hydrolyzable GTP analog GMPCPP maintain an extended lattice state while MTs in which GTP hydrolysis has occurred adopt a so-called compacted state [17,19,23,26]. These studies show that lattice compaction arises from shortening of the tubulin–tubulin inter-dimer separation as α-tubulin moves closer to the β-tubulin in the adjacent dimer in response to GTP hydrolysis.

**Figure 3:**
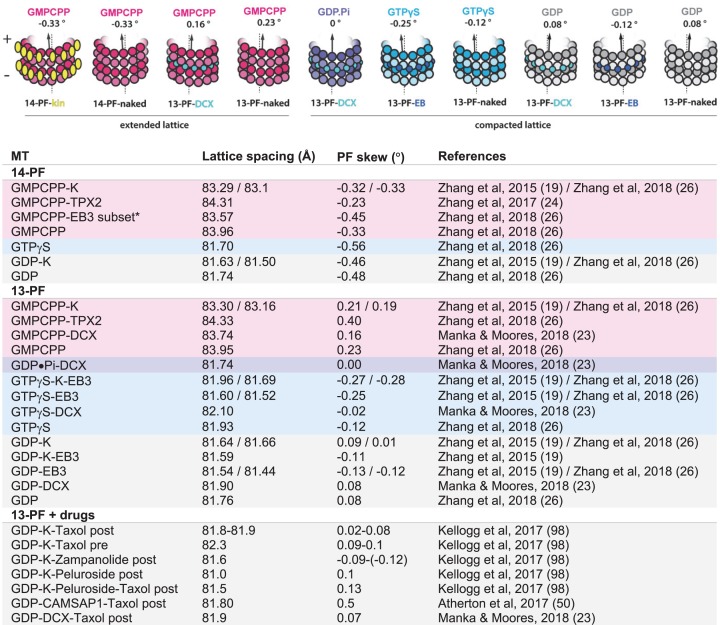
Modulation of MT-lattice conformation by MT-binding proteins and drugs Top, schematic representation of various MT lattices used in studies addressing the mechanism of dynamic instability. Bottom, table summarizing influence of PF-number, nucleotide state (color-coded along the rows) and MT-binding proteins and drugs on major MT lattice parameters: the axial repeat distance of tubulin dimers (lattice spacing) and PF skew. *EB3 induces hydrolysis of GMPCPP leading to compaction of MT lattice—only a small subset of GMPCPP-EB3 MTs has been shown to exhibit the extended lattice conformation. The impact of different drugs on MT structure depends on whether they are present during MT assembly (pre) or are added after the assembly (post). The summary is limited to mammalian tubulin due to the lack of unambiguous characterization of these parameters in yeast [80–82] and other organisms; K, kinesin-1 motor domain.

Cryo-EM structures of the MT lattice in different nucleotide states provide structural snapshots of these dynamic polymers. However, it is important to bear in mind that, to date, most of the cryo-EM MT structures considered in the context of elucidating dynamic instability mechanisms have been determined in the presence of MAP or kinesin MTBDs [17,19,23]. Furthermore, a number of these binding partners were shown to also elicit specific changes in MT lattice conformation (Figure 3). One exception is DCX, which does not appear to strongly influence MT lattice parameters in any nucleotide state (Figure 3, compare DCX-decorated and undecorated lattices). Thus DCX—with its MT-nucleating and stabilizing properties—is a valuable tool for elucidation of purely nucleotide-dependent lattice transitions underlying dynamic instability [23]. Although such studies do not show how this compaction affects lattice energetics or strain, taken together they can be used to decipher aspects of tubulin mechanochemistry and deduce the structural basis of MT dynamic instability.

### Looking for the trigger of catastrophe

Considering that tubulin dimers in the lattice are under constant mechanical strain, they can be envisioned as loaded springs that are always poised for release. Therefore, the point at which they eventually do so—driving MT depolymerization—must be a point of imbalance between the lattice binding energy and the lattice strain energy. One can think of three possible scenarios causing such imbalance: the post-hydrolysis lattice compaction may (1) weaken the lattice contacts below the threshold of resisting the existing strain, (2) increase the strain beyond the resistance threshold of the existing contacts or (3) synergize both these effects leading to lattice contacts being overcome by the strain. The first cryo-EM reconstructions of MTs of sufficient resolution (∼4-5 Å) to reveal the structural details of lattice compaction involved kinesin-decorated 14-PF lattices in the GMPCPP (GTP-like) and GDP states. Lattice contacts were still relatively poorly defined at these resolutions, but the source of MT instability was attributed to strain within the MT wall due to changes at the longitudinal inter-dimer contacts on lattice compaction. A subsequent study compared kinesin-decorated 14-PF extended GMPCPP and compacted GDP lattices with the EB-decorated 13-PF compacted GTPγS and GDP lattices at near-atomic resolution. These comparisons identified in GTPγS and GDP states a distortion of a helix (H8) in α-tubulin next to the longitudinal inter-dimer interface, compared with the GMPCPP state [19]. In addition, the compacted post-hydrolysis GTPγS and GDP structures displayed α-tubulin rotation that led the authors to conclude that GTP hydrolysis induces strain. This idea points to scenario (2), listed above, as the underlying explanation for MT instability: the post-hydrolysis lattice compaction increases the strain beyond the resistance threshold of the existing contacts.

**Figure 4:**
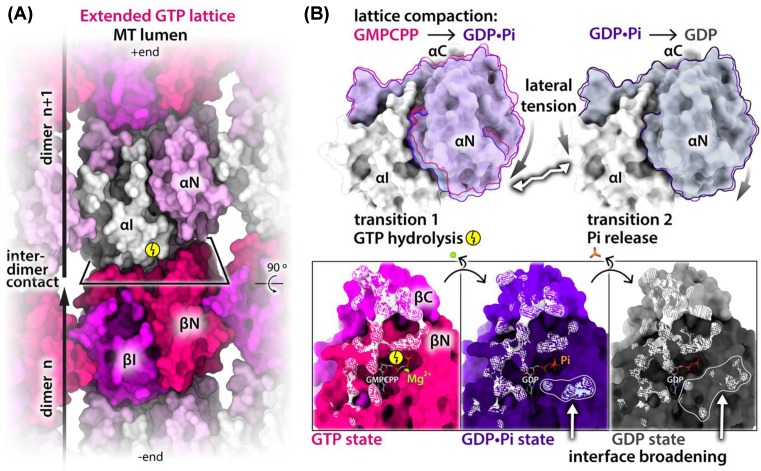
Uneven compression of α-tubulin and the resultant strengthening of longitudinal and weakening of lateral lattice contacts (**A**) MT extended lattice fragment (PDB: 6EVW) seen from the lumen and colored by tubulin subdomains: αI, βI, αN, βN–intermediate and N-terminal domains of tubulin α and β subunits, respectively. Tubulin dimer boundaries and lattice polarity are indicated, and longitudinal inter-dimer interface is framed in a perspective rectangle; C domains are not visible from this view as they project from the outside of the MT wall. (**B**) Top, αN subdomain movements after GTP hydrolysis (transition 1) and after Pi release (transition 2) relative to the αI subdomain; αN in the GDP.Pi state is semi-transparent blue and in the GDP state—gray. Outlines are included for convenient inspection of the movements; αN moves further than αI, as denoted with different sizes of the curved arrows. This causes tensions between the N and I subdomains (zigzag arrow), resulting in their loosening. Bottom, (+) end view of the β subunit’s longitudinal interface in different nucleotide states, as color-coded and shaded by subdomains; to aid viewer’s orientation, the rectangular frame is related to that in panel (A) by 90° rotation; αN moves further toward the (−) end compared with αI, as it has ample space, whereas αI extensively interacts (white shading) with the βN and βC subdomains of the preceding dimer. The GTPase (bolt symbol)-driven transitions broaden this longitudinal interface—as indicated with outlines and white arrows—through additional interactions with αN. Protein structures are shown with SES generated with ChimeraX [71]. The nucleotides are shown as white and heteroatom-colored sticks, and magnesium ion is shown as a ball.

At the time of these first high-resolution studies, there were only a few MT structures suitable for accurate cross-comparison. As a result, comparisons between inherently different MT geometries (arising from PF number variation), conflated with binding effects of disparate MT-binding partners, such as kinesins or EBs, were inherent to the approach (Figure 3), making it challenging to attribute conformational changes in the lattice specifically to the nucleotide state. Since structural transitions associated with sequential steps of the GTPase action are subtle (in the range of ∼2 Å), they ideally need to be examined in structures with a single PF architecture and binding partner. We took advantage of DCX’s ability to produce uniform 13-PF MTs, as introduced earlier, to obtain a 13-PF extended GMPCPP lattice and 13-PF compacted GDP lattice. We also developed a rapid MT polymerization and plunge freezing protocol exploiting DCX’s robust MT nucleation activity that enabled us to capture the bona fide GDP.Pi intermediate state [23]. Comparison of the DCX-stabilized structures at near-atomic resolution unveiled uneven compression of α-tubulin upon lattice transition from GTP-like (GMPCPP) to GDP.Pi state (Figure 4). Such unevenly distributed lattice compaction is dictated by the geometry of the inter-dimer longitudinal contacts, which anchor the intermediate subdomain of α-tubulin (αI), leaving the N-terminal subdomain (αN) more translational and rotational freedom. The resultant αN shift and twist toward the MT (−) end and lumen tightens the longitudinal inter-dimer interaction, while loosening the lateral connection with the more restricted αI domain from the neighboring PF. The subsequent Pi release (GDP.Pi to GDP transition) causes further loosening of homotypic inter-PF contacts—this time between the β-tubulins—and further reinforces the longitudinal lattice contacts (Figure 4). This observation supports scenario (1) above: that dynamic instability arises from the combined two-step lateral weakening and longitudinal strengthening that renders the lattice unable to counteract the strain energy, triggering MT catastrophe via well-documented PF peeling [9,83]. However, it does not exclude the third possibility (scenario (3)), that the lattice strain also increases through those transitions as previously proposed [17,19].

The study of naked MTs—not decorated with any binding partners—by the Zhang and Nogales labs [26] presents a parallel approach for separating the effects of nucleotide and MT-binding partners on MT structure. The authors focused on reconstructions without imposition of pseudohelical symmetry and highlighted the role of the seam in dynamic instability, showing its deviation from cylindrical symmetry in the GDP state, but not in the GMPCPP or GTPγS states. From the observed ∼1–3 Å relative separation of PFs at the seam, the authors inferred that it is likely to crack open in the absence of the stabilizing GTP cap, triggering MT catastrophe. No other post-hydrolysis changes in any lattice contacts were described.

The GTPγS state—previously widely accepted as a surrogate of the GDP.Pi state—appears to have mixed characteristics relative to the natural nucleotide states (Figure 3). Its tubulin dimer conformation resembles that of the GDP state, but the MT lattice shows tendency for more left-handed PF skew compared with the GDP state [23,26], similar to that of the GDP-Pi state [23] (Figure 3). This may explain preferential binding of EB proteins to GTPγS-MTs and their dynamic MT end-tracking behavior, since EBs themselves induce a left-handed skew (Figure 3) and thus appear to have higher affinity for that particular lattice geometry. On the other hand, similarity of the GTPγS-tubulin to GDP-tubulin explains poor MT nucleation in the presence of this nucleotide analog [84].

**Figure 5:**
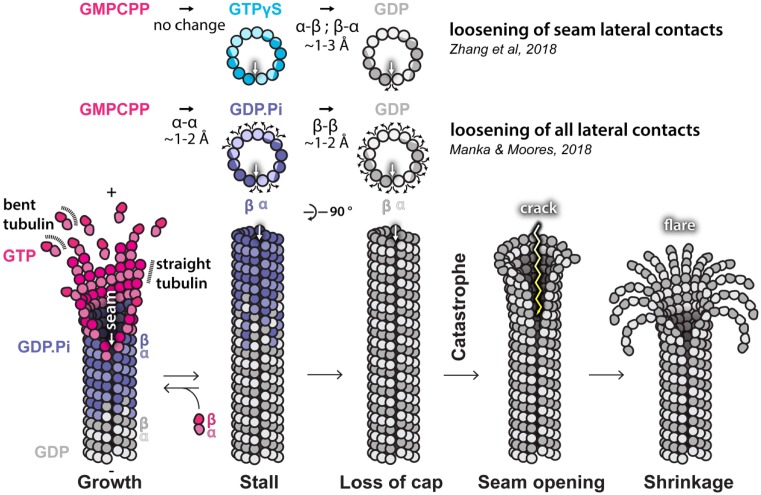
Proposed models of the structural basis of dynamic instability Recent cryo-EM studies highlight two potential structural sources of dynamic instability: (i) lateral loosening at the MT seam (white arrow) in the GDP state, but not in the GTP-like (GMPCPP) state or the GTPγS state [26] that shares some characteristics with the GDP.Pi state [23] and (ii) two-step lateral loosening between all PFs in the MT lattice, first on transition to GDP.Pi state and then to GDP state [23]. Both scenarios are depicted at the top part of the figure using schematics of MT cross-sections in transversal plane. Due to PF stagger, the cross-section perpendicular to MT axis runs across both α- (lighter) and β-tubulin (darker), enabling depiction of sequential lateral loosening between the subunits (curved arrows). These observations can be summarized in a single coherent model of MT catastrophe shown in the bottom part of the figure, as the studies agree that the seam appears to be the weakest link of the lattice.

The seam-centric structural model of MT catastrophe [26] and the more holistic model [23] are summarized in Figure 5. It is uncontroversial that the seam is the weakest link in the MT after lattice compaction due to the inevitable mismatch between heterotypic lateral contacts. The seam-centric model assumes overall increase in lattice strain after lattice compaction (scenario (2)), potentially corresponding to the greater conformational rigidity of the post-hydrolysis dimer [85] by an unknown allosteric mechanism. But what about truly helical MTs that do not have a seam? We know that *in vitro* experiments produce a range of MT architectures [8,13,86], including 15- and 16-PF helical MTs, but if dynamic instability relied solely on seam instability, it suggests that a hyperstable subpopulation of MTs would be present in dynamics assays, which has not been reported. This implies that dynamic instability does not strictly require the presence of a seam. On the other hand, a GTPase-dependent hierarchy of strength in all lateral contacts [23] can explain MT growth from GTP-tubulin (stronger lateral contacts) and the complete post-hydrolysis shrinkage (weakening of all lateral contacts) without invoking lattice strain upon compaction. These different scenarios can be computationally modeled [83,87,88], but new approaches to studying the energetic aspects of nucleotide-dependent tubulin transitions and properties in and out of MT lattice are necessary to fully unravel the remaining conundrums.

### Conservation of MT dynamics mechanisms

It is also worth to mention that MT lattice characteristics, such as those presented in Figure 3, are not universally conserved across species. Because native yeast (*Saccharomyces cerevisiae* and *Schizosaccharomyces pombe*) tubulin can be purified in relatively large quantities, the dynamics of yeast MTs have been investigated *in vitro* [89,90,81]. However, they have so far proven more challenging to study by cryo-EM structural analyses with currently available image processing and 3D reconstruction algorithms due to their heterogeneity and unusual architecture compared with mammalian tubulin. This includes more twisted PFs [80] and potentially multiple seams—this latter idea had been previously used to invoke the existence of EB-dependent yeast MTs composed of mostly or completely seam-like heterotypic (α-β, β-α) lateral contacts (so called A-lattice) [91]. Multiple seams would lead to MT reconstructions with mixed α- and β-tubulin registers [82] and thus unreliable estimates of inter- and intra-dimer spacing in the lattice. Significant progress has been made in expressing tubulin recombinantly [92–94] and in isolating sufficient quantities of tubulin from different organisms to undertake structural studies [95]. Future studies will shed light on whether the mechanisms of dynamic instability described for tubulin from mammalian brain are more generally applicable.

## Emerging mechanisms of MT stabilizing agents

**Figure 6:**
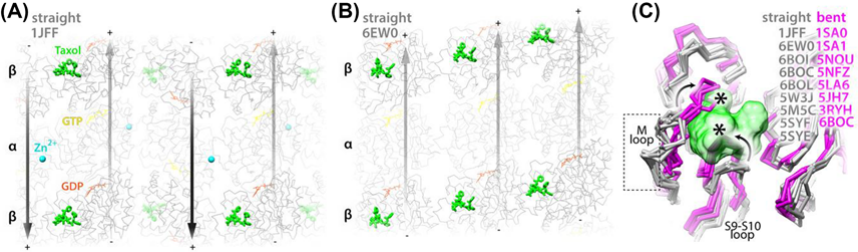
Mechanism of MT stabilization by Taxol (**A** and **B**) Taxol-bound tubulin in the straight conformation associated into PFs forming either the zinc-induced sheet lattice [100] (A) or the MT lattice (B). Each panel shows a single-layer fragment of each lattice and MT lattice (B) is viewed from the lumen. Taxol stabilizes each lattice against cold temperature depolymerization and aging. The PFs in the sheet (A) run in an anti-parallel fashion and in MTs (B) in a parallel fashion, with a stagger determining helical rise of the MT cylinder. The PF polarity is indicated with (+) and (−) signs and with the arrows running along the PF ridges (outside crests in the MT context). Tubulin backbone and nucleotides are shown as wires, Taxol as sticks, and zinc as balls (Chimera [107]); PDB structure identifiers and the color-coding of features are indicated; this depiction uses fading to indicate depth. (**C**) Close-up view on the Taxol-binding site and its perturbation upon tubulin transition from straight (gray) to bent (magenta) conformation. Multiple straight tubulin structures (with and without Taxol) are aligned on the β-tubulin subunit together with multiple bent tubulin structures, determined by cryo-EM or X-ray crystallography. Tubulins are shown with backbone stick representation and Taxol with molecular surface representation. The PDB identifiers are listed according to tubulin conformation; 6BOC can be found in both columns, since it represents a complex containing two, Taxol-bound (straight) and unbound (bent) tubulin dimers. Straight-to-bent conformational transition moves the S9–S10 loop and the M loop toward Taxol-binding pocket (curved arrows), which severely clashes with Taxol density (asterisks). Only in the straight conformation the M loop is in the position compatible with forming lateral contacts (boxed with the broken line) in both MTs and zinc-induced sheets.

Owing to their essential role in cell division, MTs have been targeted by various anti-tumor drugs. Some of them, such as vinblastine or colchicine, perturb MT polymerization and their mode of interaction with curved tubulin dimers was visualized by X-ray crystallography [96,97]. Others, such as Taxol, zampanolide and peluroside, block MT depolymerization. Recent high-resolution cryo-EM studies shed light on the distinct impact of each of these drugs on the MT lattice [98]. Taxol and zampanolide bind to the same pocket at the lumenal side of the β-subunit (Figure 6), and peluroside binds directly to lateral contacts but on the outside of the MT cylinder. MTs stabilized by Taxol or zampanolide are flexibile and characterized by slight deviations from a cylindrical shape along the MT. On the other hand, peloruside stiffens (regularizes) the lattice, eliminates the opening otherwise present at the seam in the GDP state (as discussed above) and overrides the flexibility from stabilization caused by Taxol in doubly bound MTs.

While the MT-stabilization mechanism of peluroside appears straightforward, since it relies on stabilization of lateral contacts, the mechanism of Taxol and its relatives is less obvious, and a long-running topic of debate. Some have suggested that Taxol allosterically prevents or reverses GTPase-driven lattice compaction, thus inducing the stable extended GTP-cap like lattice state [17]. This is in agreement with earlier work showing an increase in the tubulin axial repeat when MTs were formed in the presence of Taxol compared with GDP-MTs without Taxol [99]. Several subsequent cryo-EM studies have shown no or only minimal (∼0.4 Å) lattice expansion in Taxol-bound MTs [23,98] (Figure 3). Crucially, that minimal expansion was captured only when MTs were polymerized in the presence of Taxol (Figure 3, ‘pre’) and not when Taxol was added after MT polymerization (Figure 3, ‘post’). Thus, most studies now agree that Taxol binding neither requires lattice expansion nor causes it in pre-established MTs.

Another important clue about Taxol’s mechanism of MT stabilization comes from the earliest high-resolution tubulin structures solved by 2D electron crystallography of Zn^2+^-induced sheets of anti-parallel PFs of Taxol-bound tubulin, in which α/β-dimers adopt the MT-lattice-like straight conformation [14,100] (Figure 6). Taxol binding stabilizes such sheets: therefore, Taxol may have a role in stabilizing this straight conformation of tubulin, as also reported elsewhere [101], and this could be the basis of its MT-stabilizing mechanism. This idea is consistent with many X-ray structures of bent tubulin, showing profound conformational change in the M-loop (the major contributor to the lateral lattice contact) and the S9–S10 loop, lining the Taxol-binding site. The straight-to-bent transition of tubulin moves the M-loop away from the MT lateral contact and the S9–S10 loop inside the Taxol-binding pocket, occluding it (Figure 6). No Taxol-bound structures of bent tubulin have been reported; this reinforces the idea that bound Taxol sterically prevents the straight-to-bent transition and thus prevents PF peeling, thereby keeping the β-M-loops in place and stabilizing lateral lattice contacts. The enhanced lattice flexibility upon Taxol binding [102] is consistent with post-hydrolysis lateral loosening of the MT lattice [23]—Taxol binding does not tighten the lateral contacts, but rather holds every other M-loop along the lattice in the lattice-like conformation. The lattice heterogeneity and potential trapping of MTs in some distinct intermediate state when Taxol is present during MT polymerization may be important in reducing the affinity of certain MAPs, like DCX, for such MTs [23,103]. Interestingly, another MT-stabilizing drug, epothilone, binds at a similar site to Taxol [104], but it can also bind to bent tubulin [105,106]. Thus, different MT-stabilizing drugs may exploit different mechanisms to prevent MT depolymerization even while binding in the same site on tubulin.

## Beyond the lattice: MT end structures

Much of the forgoing work aimed at addressing the GTPase-linked structural transitions of tubulin at as high-resolution as possible, has focused on the MT lattice, the regularity of which makes it highly suitable to be studied using so-called single particle cryo-EM structural averaging approaches [108]. In contrast, the ends of MTs—where key structural transitions that correspond to dynamic events also take place—are very heterogeneous, and it is therefore challenging to access their 3D structure or structures. Early cryo-EM 2D micrographs clearly demonstrated this structural heterogeneity *in vitro* [9], while revealing a correlation between the overall phase of MT dynamics and the appearance of MT ends: the ends of MTs captured under assembly conditions showed evidence of PFs finishing unevenly in otherwise cylindrical-appearing MTs, while the ends of disassembling MTs demonstrated elaborately curling and apparently well-separated PFs. Later studies also described the presence in growth-promoting conditions of—sometimes very long—sheets of tubulin PFs, gently curving away from the MT axis while retaining lateral connectivity between PFs [109]. These evocative and compelling 2D images have informed decades of discussions and models concerning dynamic instability. Importantly, they emphasize that the MT end does not correspond to a single position or tubulin structure, but is rather a series of zones that correspond to sets of conformational transitions that capture aspects of dynamic instability.

Cryo-electron tomography offers an unbiased route to determining 3D structures—albeit at lower resolutions— without averaging [110]. MT ends are thus an ideal target and several groups have begun to examine MT ends in 3D by cryo-ET [111]. Visualization of MTs in 3D immediately provides a greater appreciation of the complexity of their structures and largely supports previous concepts derived from 2D images: that at least some extended sheets are seen at growing or stable MT ends, while PFs separate and curl away from depolymerizing ends [20,50,112,113]. However, a very recent and comprehensive study comparing MT ends from a variety of sources *in vitro* and *in vivo* and using both cryo-EM and freeze-substituted EM samples has reached a very different conclusion: PFs at the ends of all MTs—growing or shrinking—are all curved and not laterally connected [83]. Thus, the molecular structure of MT ends remains a topic of active debate. Even if a consensus is reached concerning their quaternary organization, without the structural resolution that comes from averaging, it is not currently possible to visualize the nucleotide state of the tubulin in each region of the MT end. Thus, a central and ongoing challenge for the future will be connecting the structure of MT ends to phases of dynamic instability and to the biochemical state of tubulin. This in turn has wide implications for the regulation of MTs by their cellular binding partners.

## Conclusion

For all aspects of MT biology, the snapshots of these polymers captured using cryo-EM will continue to provide critical insights into the fundamental mechanisms of their dynamics, the way in which they are regulated by cellular components, and the way in which MTs are recruited to many cellular functions in diverse physiological contexts.

## Summary

- Cryo-electron microscopy allows microtubules to be captured and imaged in their solution-like state.
- This approach has provided significant insight into their dynamic mechanisms and interactions with cellular binding partners.
- High-resolution 3D structure determination of cryo-EM microtubule images using single particle image processing has allowed the binding sites of numerous microtubule-associated proteins and microtubule-stabilizing drugs to be precisely characterized.
- Low-resolution 3D structure determination using cryo-electron tomography has begun to reveal conformational heterogeneity of microtubule ends but mechanistic consensus remains lacking.

## Abbreviations

AAA: ATPases associated with diverse cellular activities
CAMSAP: calmodulin-regulated spectrin-associated protein
CH: calponin homology
Cryo-EM: cryo-electron microscopy
DCX: doublecortin
EB: end-binding protein
EM: electron microscopy
MAP: MT-associated protein
MT: microtubule
MTBD: MT-binding domain
PF: protofilament
PRC1: protein regulator of cytokinesis 1
SES: solvent-excluded surface
TPX2: targeting protein for Xklp2 (Xenopus kinesin-like protein 2)
WHAMM: WASP (Wiskott-Aldrich syndrome protein) homolog-associated protein with actin, membranes and microtubules
